# Epidemiological analysis, risk factors, and spatial-temporal clustering of classical swine fever virus in China

**DOI:** 10.1186/s40813-026-00486-5

**Published:** 2026-01-21

**Authors:** Wenchao Gao, Qingyuan Liu, Jiansheng Zhou, Xiaoxue Jiang, Qing Wang, Yuntong Shi, Xiaomin Liu, Xia Liu, Lili Wu, Qin Zhao, Xiaowen Li

**Affiliations:** 1 College of Agriculture and Biology, Liaocheng University, Liaocheng, 252059 China; 2 Shandong Engineering Laboratory of Pig and Poultry Healthy Breeding and Disease Diagnosis Technology, Xiajin New Hope Liuhe Agriculture and Animal Husbandry Co., Ltd., Dezhou, Shandong 253200 China; 3 Shandong Animal Health Technology Center, Jinan, Shandong 250100 China; 4 College of Veterinary Medicine, Hebei Agricultural University, Baoding, 071000 China; 5 Department of Preventive Veterinary Medicine, College of Veterinary Medicine, Northwest A&F University, Yangling, Shanxi 712100 China

**Keywords:** Classical swine fever virus, Pathogen prevalence, Epidemiological investigation, Risk factors, Spatiotemporal clustering, China

## Abstract

**Background:**

Classical Swine Fever (CSF), or swine cholera, is a highly acute, febrile, and contagious disease caused by the Classical Swine Fever virus (CSFV) in pigs.

**Results:**

A total of 205,622 samples were collected from 689 pig farms defined as individual production units nationwide across 20 Chinese provinces/cities from January to December 2022. The results showed that at the sample level, the prevalence of CSFV was 0.25% (95% confidence interval (CI) 0.22% − 0.27%). At the pig farm level, the prevalence of CSFV was 5.08% (95% CI: 3.44% − 6.72%). The risk factors for CSFV infection in pig farms included geographic distribution, quarter, and herd category variables. Eight different temporal groups with a high prevalence of CSFV were also found in China, with the highest prevalence occurring from August to September 2022. Tissue samples and oropharyngeal/nasal swabs showed superior detection rates (1.00% and 0.34%, respectively) and are thus recommended for CSFV surveillance.

**Conclusions:**

In conclusion, this data complements the existing studies on the Chinese CSFV positive rate, risk factors, and temporal clustering. Our findings identify high-risk periods (August – September 2022) and regions (Northwest China), providing targeted strategies for CSFV control.

## Background

 Classical swine fever is a highly acute, febrile, and contagious disease caused by the classical swine fever virus (CSFV) in pigs [1]. The disease is distributed worldwide and is one of the most destructive infectious diseases in the pig industry [1, 2]. CSFV is a positive-stranded enveloped RNA virus with a diameter of 40–60 nm and a genome length of 12.3 kb [3]. CSFV is typically genotyped based on its E2 glycoprotein, which is divided into three main genotypes, each consisting of three or four subtypes (including 1.1, 1.2, 1.3, 1.4; 2.1, 2.2, 2.3; and 3.1, 3.2, 3.3, 3.4) [4–6]. The virus has been isolated in China, but mainly genotype 2 has been reported [1, 4, 6, 7].

Many countries and regions, such as Australia, North America, and Western Europe, have successfully eradicated CSFV. However, there are still many regions where it remains endemic [8, 9]. Reports indicate that CSFV is currently widespread in parts of Asia, Africa, and Central and South America [7, 10, 11]. The clinical manifestations of CSFV infection in pigs categorize the disease into acute, subacute, chronic, and congenital types. Pigs infected with CSFV may show symptoms like high fever (above 40℃), anorexia, conjunctivitis, respiratory issues, miscarriage, and death. However, these clinical symptoms are not specific [1, 12, 13]. The most commonly used laboratory diagnostic method for detecting CSFV is RT-PCR, known for its high specificity, sensitivity, and speed [14, 15]. Since the duration of CSFV viremia is brief, antibody detection methods are also frequently employed [16]. In China, the primary method to prevent and control CSFV is through the use of live attenuated or subunit vaccines targeting the C strain [17–20].

China is the largest producer and consumer of pork products, and controlling and eradicating swine fever is crucial for the development of the pig industry [2]. Therefore, from January to December 2022, 205,622 clinical samples were collected from 20 provinces and cities in China to investigate the prevalence of CSFV and analyze the factors that influence the CSFV prevalence across China. This study aimed to: (1) quantify CSFV prevalence at individual-animal and farm levels; (2) identify spatial-temporal clusters; and (3) evaluate risk factors influencing CSFV distribution in China during 2022.

## Methods

### Study area and population

All samples were collected from 19 provinces and a city in China, which have the most developed pig industry. A total of 205,622 samples were collected from 128 companies integrated farming corporations and 689 pig farms individual production units under these companies. Farms were selected based on geographical representation and operational scale to minimize bias. The pig farms are distributed across six regions: Northeast China (Heilongjiang and Liaoning Province), Central South China (Henan, Hubei, Hunan, Guangdong, and Guangxi Province), East China (Jiangsu, Anhui, and Shandong), Northwest China (Gansu and Shaanxi), North China (Hebei, Inner Mongolia, Shanxi, and Tianjin), and Southwest China (Guizhou, Yunnan, and Sichuan). The geographical coordinates of pig farms were obtained using Baidu Map (https://map.baidu.com/).

The breeding farms, with breeding stock ranging from 750 to 3000 sows, all maintained a consistent herd composition. This included pre-weaning piglets (0–21 days old), gilts (90–230 days old), adult sows (over 230 days old, either in gestation or with a gestation history), and boars (over 300 days old). The fattening farms included nursery piglets (21–70 days of age) and growing-finishing pigs (70–180 days of age), with a production capacity of over 6,000 pigs.

All adult sows and boars were vaccinated three times a year with live CSF vaccines (C strain from PULIKE BIOLOGICAL ENGINEERING, INC.) or CSF subunit vaccines (Tecon Biology Co., Ltd). All fattening pigs were vaccinated with live CSF vaccines at the age of 35 days or 56 days and were boosted with live CSF vaccines after 21 days post-vaccination. All gilts were vaccinated with live CSF vaccines at the age of 140 days and 210 days.

### Sampling design and sample collection

Due to the biosecurity and epizootic prevention and control measures implemented on pig farms, a convenience sampling plan was conducted for this investigation. This study is classified as a prevalence study because it aims to quantify the proportion of CSFV-positive individuals and farms within a defined population (pigs from 20 provinces and cities in China) during a specific time frame (January to December 2022). Additionally, it seeks to identify spatiotemporal clustering and evaluate associated risk factors, which are key objectives of prevalence studies. The findings provide baseline data for CSFV control and prevention strategies. The sampling parameters are clearly specified as follows: (1) geographical representation, encompassing six major pig-producing regions in China; (2) farm scale, including breeding farms with 750 to 3,000 sows and fattening farms with an annual output of ≥ 6,000 pigs; (3) herd category, covering all production stages, such as pre-weaning piglets, nursery pigs, growing-finishing pigs, gilts, sows, and boars; and (4) clinical relevance, with samples exclusively collected from pigs exhibiting suspected CSFV symptoms, including fever, anorexia, miscarriage, and diarrhea—to ensure targeted representation of potential cases. Samples were exclusively collected from pigs exhibiting clinical symptoms such as miscarriage, fever, anorexia, weight loss, diarrhea, and death to ensure targeted representation of suspected cases. Samples included blood, oropharyngeal and nasal swabs, tissues, semen, blood from the umbilical cord, placenta, testicular fluid, feces, and environmental swabs. Tissue samples, including tissues, semen, and placenta, were mixed with sterile physiological saline, homogenized, and subjected to three cycles of freezing and thawing. The supernatants were collected after being centrifuged at 10,000 × g for 5 min. Blood samples were centrifuged at 3,000 × g for 5 min to obtain serum. Other samples, such as oropharyngeal and nasal swabs, testing fluid, feces, and environmental swabs, were also included. First, a small amount of sterilized physiological saline was added and mixed well. Centrifugation was done at 10,000 rpm for 5 min, and the supernatant was collected. The samples were stored at -20 °C until use.

### Detection of CSFV by real-time fluorescence quantitative polymerase chain reaction

According to the manufacturer’s instructions, commercial kits (Nanjing Vazyme Biotech Co., Ltd, China) were used to extract viral RNA from the samples. The extracted RNA was used as a template to detect CSFV by RT-qPCR (Nanjing Vazyme Biotech Co., Ltd, China). All primers used in this study are summarized in Table 1. The RNA was amplified using the RT-qPCR one-step kit: reverse transcription was performed at 50 °C for 15 min, followed by pre-denaturation at 95 °C for 30 s. Subsequently, denaturation was carried out at 95 °C for 10 s, followed by annealing at 60 °C for 30 s. This cycle was repeated a total of 45 times.

**Table 1:** Primers used in this study

Primer	sequence (5’ to 3’)	Length (bp)
CSF-F	TGCCCAYAGTAGGACTAGCA	20
CSF-R	GTCGAACTACTGACGACTG	19
CSF-P	TAGTGGCGAGCTCCCTGGGTG	21

### Data analysis

All collected data were extracted and organized in Excel (Excel 2021, Microsoft, USA). The associations between the positive rate of CSFV and various factors, such as regions, sample types, quarters, and pig herds, were analyzed using the Chi-square test in SPSS 26.0 software (IBM, Chicago, IL, USA). The 95% confidence intervals (CIs) were investigated. Meanwhile, P-values less than 0.05 were considered statistically significant.

The trend analysis of the CSFV positive rate was conducted following these steps. A pig farm or company with at least one CSFV positive sample was classified as positive for CSFV. The CSFV status of a pig farm was considered a dichotomous variable, either positive or negative for CSFV. Spatio-temporal scanning was carried out using the Poisson distribution model. Data from companies testing positive for CSFV were analyzed spatially and temporally using SaTScan V10.1 software. The scanning period ranged from January 1, 2022, to December 31, 2022, with a monthly interval. The maximum aggregation space and time were set at 50% of the study period, and 999 Monte Carlo simulations were executed. If the P-value of the log-likelihood ratio (LLR) test is below 0.05, the region is considered to exhibit aggregation. Furthermore, maps were generated using ArcGIS 10.7 software (ESRI, USA).

## Results

### Trends in the positive rate of CSFV

**Fig. 1:**
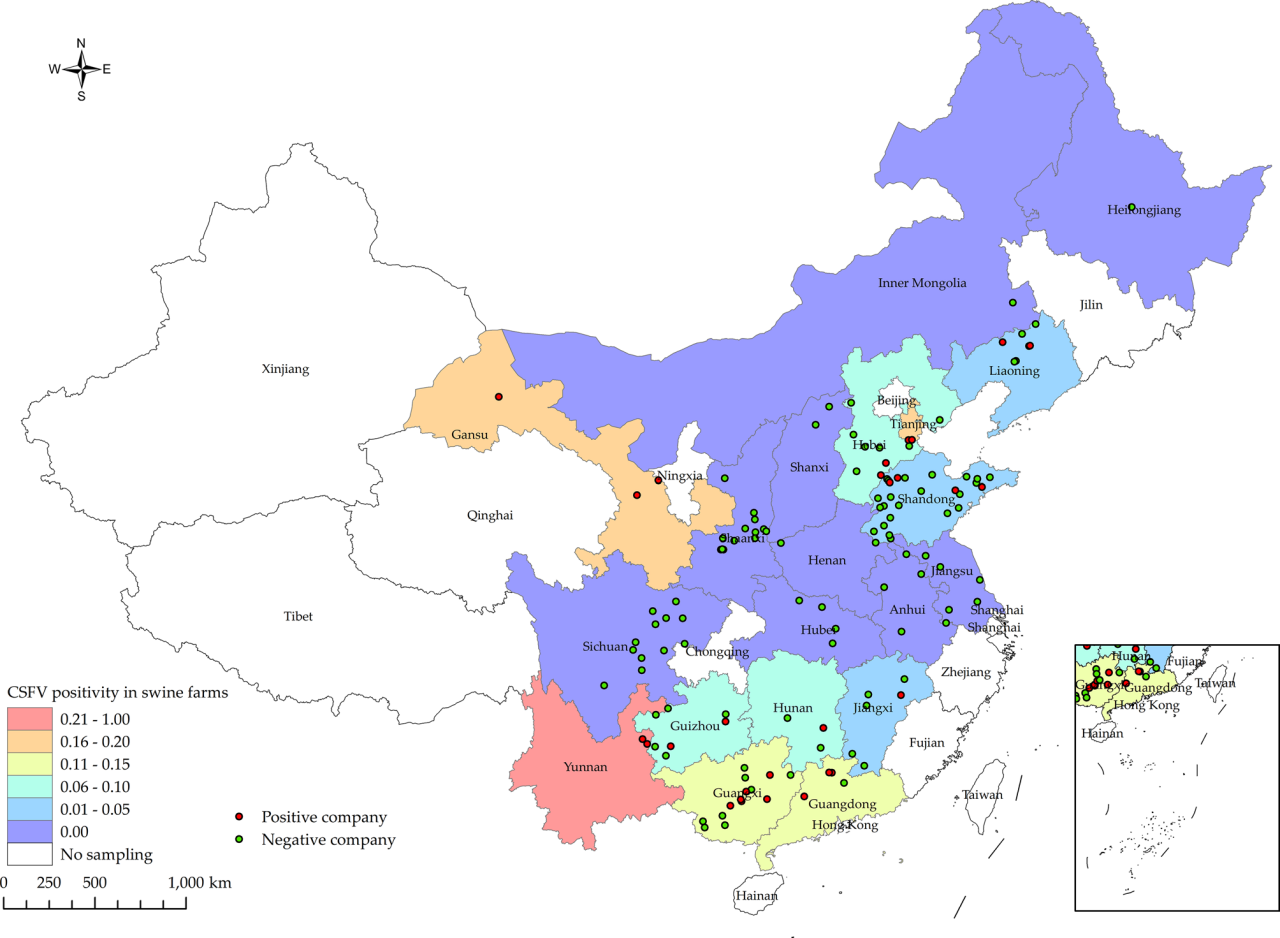
Distribution of CSFV positive rates at the farm level for samples collected between January 2022 and December 2022 in different provinces and cities in China, along with their respective collection locations. Different colored boxes represent positive rates in provincial, with green dots indicating CSFV-negative companies and red dots indicating CSFV-positive companies

A total of 205,622 samples were obtained from 19 provinces and 1 city in China from January to December 2022. The distribution map of the samples is shown in Fig. 1. The Classical Swine Fever Virus (CSFV) positive rates in provinces or cities are described in Table 2. The overall positive rate of samples and farms for CSFV was 0.25% (95% CI: 0.22%-0.27%) and 5.08% (95% CI: 3.44%-6.72%), respectively. The highest prevalence of samples and farms in Yunnan Province was 8.71% (95% CI: 6.01%-11.40%) and 100% (95% CI: 0–0), respectively. Additionally, the positive rate of samples and farms in Gansu Province is 1.44% (95% CI: 1.26%-1.63%) and 15.79% (95% CI: 3.64%-27.94%), respectively. The positive rate of samples in other provinces is low or even 0%.

From the various samples in Table 3, the tissue samples had the highest positive rate at 1.00% (95% CI: 0.77%-1.23%), followed by 0.34% (95% CI: 0.28%-0.40%) for oropharyngeal and nasal swabs. These sample types showed superior detection rates and are thus recommended for CSFV surveillance. The results of the Pearson Chi-Square Test indicated significant differences among the sample types.

**Table 2:** Prevalence of CSFV in selected provinces and cities in China

Provinces	No. of positive samples/province	Total samples/Province	Prevalence with 95% CI (%)	No. of positive farms	No. of farms/Province	Prevalence with 95% CI (%)
Heilongjiang	0	559	0(-)	0	5	0(-)
Liaoning	20	14,381	0.14(0.08–0.20)	2	60	3.33(0-8.01)
Tianjing	10	2742	0.36(0.14–0.59)	2	13	15.38(0-38.08)
Hebei	2	4100	0.05(0.00-0.12)	2	31	6.45(0-15.61)
Shanxi	0	184	0(-)	0	8	0(-)
Inner Mongolia	0	3110	0(-)	0	9	0(-)
Shandong	84	37,079	0.23(0.18–0.27)	6	163	3.68(0.76–6.60)
Jiangsu	0	3444	0(-)	0	11	0(-)
Anhui	0	13,427	0(-)	0	25	0(-)
Jiangxi	6	8538	0.07(0.01–0.13)	1	33	3.03(0-9.20)
Gansu	232	16,097	1.44(1.26–1.63)	6	38	15.79(3.64–27.94)
Shaanxi	0	13,494	0(-)	0	53	0(-)
Guizhou	26	8402	0.31(0.19–0.43)	3	36	8.33(0-17.82)
Sichuan	0	13,066	0(-)	0	48	0(-)
Yunnan	37	425	8.71(6.01–11.40)	2	2	100.00(-)
Henan	0	4605	0(-)	0	15	0(-)
Hubei	0	6774	0(-)	0	29	0(-)
Hunan	10	5629	0.18(0.07–0.29)	1	18	5.56(0-17.28)
Guangdong	18	11,216	0.16(0.09–0.23)	3	25	12.00(0-25.69)
Guangxi	61	38,350	0.16(0.12–0.20)	7	67	10.45(2.93–17.97)
Total	506	205,622	0.25(0.22–0.27)	35	689	5.08(3.44–6.72)

CI, confidence interval

**Table 3:** Descriptive statistics and pearson chi-square test of the prevalence of CSFV in different sample types

sample types	No. of positive samples	No. of total samples	Prevalence with 95% CI (%)	χ^2^ values	*p*
Blood	300	145,099	0.21(0.18–0.23)	223.90	0.000^***^
Oropharyngeal and nasal swabs	121	35,110	0.34(0.28–0.40)		
Diseased tissues	74	7407	1.00(0.77–1.23)		
Semen	0	7295	0.00(-)		
Umbilical cord blood	8	4114	0.19(0.06–0.33)		
Placenta	3	3352	0.09(-0.01–0.19)		
Testicular fluid	0	2425	0.00(-)		
Feces	0	475	0.00(-)		
Environmental swabs	0	345	0.00(-)		

*p < 0.05 indicates a significant correlation between the influencing factor and CSFV prevalence; **p < 0.01; ***p < 0.001. CI, confidence interval. Note: Detection rates varied significantly by sample type (χ²=223.90, *p*<0.001), with tissues and oropharyngeal/nasal swabs showing the highest positivity. “Diseased tissues” refer to tissue samples (e.g., spleen, lymph nodes) collected from pigs showing clinical signs of CSFV infection

### Factors influencing changes in CSFV positive rates

Regions, quarters, pig herds, and sample types variables are significantly associated with the CSFV positive rates of samples (p-value < 0.05) in the Pearson Chi-Square Test presented in Table 4. These variables are all included in the multivariable logistic analysis.

**Table 4:** Descriptive statistics and pearson chi-square test for sample-level variables associated with swine fever virus positive in selected provinces and cities in China, 2022

Variables	Category	No. of positive samples	No. of total samples	Prevalence with 95% CI (%)	χ^2^ values	*p*
Regions	Northeast China	20	14,940	0.13(0.08–0.19)	425.551	0.000^***^
	North China	12	10,136	0.12(0.05–0.19)		
	East China	90	62,488	0.14(0.11–0.17)		
	Northwest China	232	29,591	0.78(0.68–0.88)		
	Southwest China	63	21,893	0.29(0.22–0.36)		
	Central South China	89	66,574	0.13(0.11–0.16)		
Quarters	Q1	72	25,908	0.28(0.21–0.34)	113.654	0.000^***^
	Q2	40	43,508	0.09(0.06–0.12)		
	Q3	266	67,450	0.39(0.35–0.44)		
	Q4	128	68,756	0.19(0.15–0.22)		
Pig herds	Boars	8	17,263	0.05(0.01–0.08)	702.542	0.000^***^
	Sows	82	35,942	0.23(0.18–0.28)		
	Piglets	24	32,813	0.07(0.04–0.10)		
	Nursery pigs	81	6012	1.35(1.06–1.64)		
	Fat pigs	233	34,067	0.68(0.60–0.77)		
	Gilts	78	79,525	0.10(0.08–0.12)		
Sample types	Main types ^a^	495	187,121	0.26(0.27–0.29)	27.511	0.000***
	Other types ^b^	11	17,995	0.06(0.02–0.10)		

*p < 0.05 represents variables associated with CSFV positivity; **p < 0.01; *** *p* < 0.001. Sample types: According to the sequence for collecting clinical samples from farm pigs. a, Main types include blood samples, oropharyngeal and nasal swabs, and diseased tissues. b, Other types include semen, umbilical cord blood, placenta, testicular fluid, feces, and environmental swabs

From the results of the multivariable logistic analysis in Table 5, it can be seen that the variable “Northwest China (0.78%, 95% CI: 0.68%-0.88%)” is significantly higher than “Northeast China (0.13%, 95% CI: 0.08%-0.19%)” with odds ratio (OR) values of 5.66 (95% CI: 3.54–9.05). Northwest China thus had significantly higher odds of CSFV detection than Northeast China. The prevalence of CSFV in samples collected in the third quarter (Q3) (0.39%, 95% CI: 0.35%-0.44%) was significantly higher than that in the first quarter (Q1) (0.28%, 95% CI: 0.21%-0.34%) with OR values of 2.66 (95% CI: 2.03–3.48). At the same time, the second quarter (Q2) (0.09%, 95% CI: 0.06% − 0.12%) was significantly lower than the first quarter (Q1), with OR values of 0.55 (95% CI: 0.37–0.814). OR values for Sows, Nursery pigs, and Fat pigs were 4.54 (95% CI: 2.17–9.48), 13.29 (95% CI: 6.30–28.02), and 11.59 (95% CI: 5.63–23.88), respectively, indicating a significantly higher prevalence of CSFV compared to boars.

**Table 5:** Multivariable logistic analysis of swine fever virus positivity and associated factors in 20 provinces and cities in China in 2022

Variables	Category	OR with 95% CI	*p*
Regions	Northeast China	1(Reference)	
	North China	0.836(0.406–1.722)	0.627
	East China	0.827(0.505–1.354)	0.450
	Northwest China	5.663(3.544–9.050)	0.000^***^
	Southwest China	1.549(0.930–2.581)	0.093
	Central South China	1.101(0.673–1.802)	0.702
Quarters	Q1	1(Reference)	
	Q2	0.551(0.372–0.814)	0.003^**^
	Q3	2.659(2.030–3.482)	0.000^***^
	Q4	1.369(1.011–1.854)	0.042^*^
Pig herds	Boars	1(Reference)	
	Sows	4.536(2.171–9.475)	0.000^***^
	Piglets	0.885(0.395–1.984)	0.766
	Nursery pigs	13.286(6.300-28.021)	0.000^***^
	Fat pigs	11.591(5.627–23.878)	0.000^***^
	Gilts	1.542(0.732–3.251)	0.255
Sample types	Mains ^a^	1(Reference)	
	Others ^b^	0.576(0.307–1.078)	0.085

*p < 0.05 indicates a significant correlation between the influencing factor and CSFV prevalence; **p < 0.01. Quarters: Q1 spans from January to March, Q2 from April to June, Q3 from July to September, and Q4 from October to December. Sample types: a, Main types include blood samples, oropharyngeal and nasal swabs, and diseased tissues. b, Other types include semen, umbilical cord blood, placenta, testicular fluid, feces, and environmental swabs

**Table 6:** Spatio-temporal clustering of CSFV prevalence in China in 2022

Cluster	Coordinates	Cluster radius (km)	Time range (yr/mo/day)	Relative risk	Log likelihood ratio	*p*
1	37.074554 N, 104.895751 E	0	2022/8/1–2022/12/31	113.51	486.78	< 0.001^***^
2	37.159509 N, 116.683233 E	0	2022/9/1–2022/9/30	169.60	216.43	< 0.001^***^
3	26.201712 N, 104.112887 E	0	2022/7/1–2022/7/31	408.28	139.60	< 0.001^***^
4	26.986048 N, 108.206119 E	0	2022/9/1–2022/9/30	19.93	42.49	< 0.001^***^
5	24.923471 N, 109.132353 E	160.60	2022/5/1–2022/6/30	7.05	26.90	< 0.001^***^
6	26.703440 N, 113.020836 E	0	2022/2/1–2022/2/28	18.18	19.46	< 0.001^***^
7	24.711952 N, 113.303992 E	0	2022/11/1–2022/11/30	10.56	15.87	< 0.001^***^
8	23.208801 N, 108.439951 E	0	2022/8/1–2022/8/31	22.06	12.80	< 0.01^**^

**p < 0.01 indicates that the incidence of CSFV in this region during a specific period was significantly different from that in other regions and timeframes; ***p < 0.001

### Spatio-temporal clustering analysis of high prevalence rates of CSFV

**Fig. 2:**
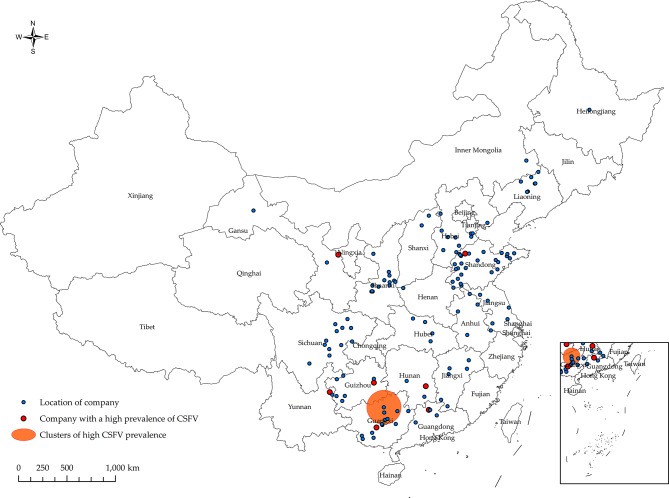
Significant spatial clustering (*p* < 0.05) of CSFV hyper-prevalence was observed in China from January 2022 to December 2022, with a maximum window size of 50% of the at-risk population. Except for the 5th, which is specific to a particular region, the remaining 7 are for a single company

Figure 2 and Table 6 present eight clusters of CSFV hyper-prevalence identified between January 2022 and December 2022 in China. Only the 5th cluster is region-specific, while the other 7 clusters correspond to individual companies. The 5th cluster was located at latitude 24.923471 N, longitude 109.132353 E, covering an area of 160.60 km². It occurred from May 1, 2022, to June 30, 2022, with a relative risk value of 7.05 and a log-likelihood ratio (LLR) value of 26.90 (*p* < 0.001).

**Fig. 3:**
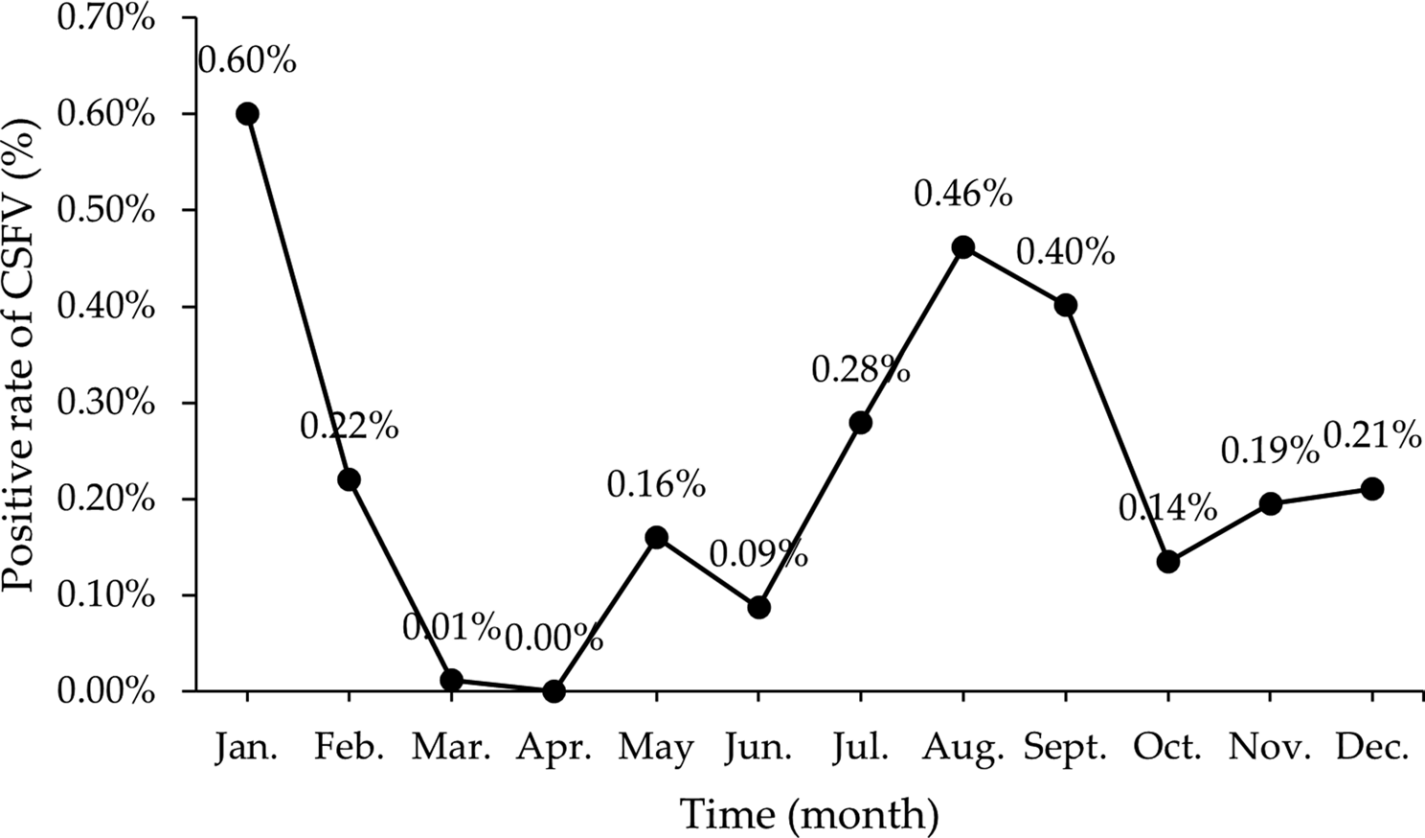
Monthly distribution of CSFV-positive samples in 2022. The x-axis represents months (January through December). The y-axis shows the positivity rate (%)

Meanwhile, Fig. 3 and the temporal scan indicate a high positive rate of CSFV during August 2022 to September 2022 (relative risk = 2.74, log-likelihood ratio = 58.37, *p* = 0.001).

## Discussion

CSFV infection exhibits a range of clinical manifestations. Acute cases are characterized by persistent high fever (> 40℃), anorexia, conjunctivitis, respiratory distress, and miscarriage in pregnant sows. Subacute and chronic cases exhibit emaciation, diarrhea, and growth retardation. Notably, some infections are asymptomatic (latent), contributing to underdiagnosis in clinical practice [21, 22]. Prevention and control of CSFV rely heavily on vaccination, biosecurity measures, and movement restrictions. In China, a national CSF control program is implemented, which includes mandatory vaccination with the C-strain vaccine, surveillance, and outbreak management [23]. These measures are essential to reduce viral circulation, prevent economic losses, and facilitate eventual eradication. Currently, due to the impact of African swine fever, many pig farms in China have experienced reduced vaccine efficacy and an increased frequency of replacing productive sows [24]. Moreover, the clinical symptoms of classical swine fever are not easily distinguishable, which can lead to inadequate diagnosis of the disease to some extent. Therefore, it is very important to understand the positive rate and the risk factors of classical swine fever.

This study collected 205,622 samples from 20 provinces and cities between January 2022 and December 2022. The overall positive rate of the samples is 0.25% (95% CI: 0.22% − 0.27%), while the positive rate specifically for the pig farms is 5.08% (3.44% − 6.72%). At the individual level, CSFV infection causes morbidity, mortality, and decreased productivity. At the farm level, outbreaks lead to economic losses due to treatment expenses, culling, trade restrictions, and diminished herd performance [25]. The positive rates of samples in Yunnan and Gansu provinces are the highest, while the positive rates in other provinces are relatively low. A low prevalence indicates effective control measures, including consistent vaccination, surveillance, and prompt outbreak response, all supported by national policies. This finding is consistent with the majority of reports. Slight differences may be due to variations in sampling time and quantity [26–32].

The detection rates of different sample types also vary. According to the results of this study, the detection rates of blood, oropharyngeal and nasal swabs, and tissue samples are higher than those of other sample types. This indicates that in clinical practice, blood, oropharyngeal and nasal swabs, and tissue samples should be prioritized as the primary samples for pigs with suspected symptoms due to their superior detection rates. Infected animals may exhibit symptoms such as high fever, anorexia, lethargy, conjunctivitis, respiratory distress, diarrhea, and neurological signs. In pregnant sows, CSFV can cause abortions, stillbirths, and congenital tremors in piglets [22, 25].

From the results of multivariable logistic analysis, it can be seen that the variables “Regions”, “Quarters” and “Pig herds” have statistical significance in relation to the positive rate of CSFV. The relatively high CSFV positive rates in the “Northwest Region” and the “Third Quarter (Q3)” indicate that attention should be paid to intensifying swine fever vaccination in the Northwest Region and the Third Quarter. The higher prevalence observed in the Northwest Region may be attributed to factors such as lower vaccination coverage, regional climate conditions, a higher density of smallholder farms, or the presence of wildlife reservoirs. The increased prevalence in Q3 (July – September) could be linked to seasonal factors such as temperature, humidity, or management practices during this period [33]. The risk of infection is higher in “Nursery pigs” and “Fattening pigs” compared to other pig populations. It is important to also consider the effectiveness of vaccine immunization, as it is related to the positive rate of CSF antibodies reported in previous studies on pig populations at this stage [33].

The results of the spatiotemporal clustering analysis revealed eight clusters of CSFV hyper-prevalence identified between January 2022 and December 2022 in China. Only the 5th group is specific to a particular region, while the remaining 7 groups represent individual clusters. The results indicate that the prevalence of CSFV is mainly regional and localized, concentrated in the central-south, southwestern, and western regions [28, 30, 33].

## Conclusion

In summary, this nationwide study revealed a low prevalence of CSFV in China during 2022, characterized by spatial and temporal clustering. Northwest China and the third quarter (Q3) represent high-risk regions and periods for CSFV transmission. Targeted surveillance using tissue samples and oropharyngeal or nasal swabs, combined with enhanced vaccination and biosecurity measures in high-risk areas and seasons, is recommended to further reduce CSFV transmission. These findings contribute to evidence-based control strategies and support ongoing national efforts toward CSF eradication.

## Data Availability

All data generated or analyzed during this study are included in this published article.

## Abbreviations

CSF: Classical Swine Fever
CSFV: Classical Swine Fever virus
RT-qPCR: Real-time quantitative polymerase chain reaction
CI: Confidence interval
OR: Odds ratio
LLR: Log-likelihood ratio
